# Biological Augmentation of Meniscal Repair: A Review with Insights into Injectable Hydrogel Delivery

**DOI:** 10.3390/gels11100786

**Published:** 2025-10-01

**Authors:** Marta Tuszynska, Joanna Skopinska-Wisniewska, Anna Bajek

**Affiliations:** 1 Department of Oncology, Ludwik Rydygier Collegium Medicum in Bydgoszcz Nicolaus Copernicus University in Torun, Lukasiewicza 1 St., 85-821 Bydgoszcz, Poland; a.bajek@cm.umk.pl; 2 Laboratory for Functional Polymeric Materials, Department of Chemistry, Nicolaus Copernicus University in Torun, Gagarina 7 St., 87-100 Torun, Poland

**Keywords:** hydrogels, meniscus, osteoarthritis, regenerative medicine, meniscus tears, platelet-rich plasma

## Abstract

Meniscal injuries are common and often lead to chronic pain, joint instability, and an increased risk of osteoarthritis. Traditional treatments, such as partial meniscectomy, may accelerate joint degeneration. In recent years, biologically active therapies, including platelet-rich plasma (PRP), mesenchymal stem cells (MSCs), hyaluronic acid (HA), bone marrow aspirate concentrate (BMAC), collagen, growth factors (GFs), and silk fibroin (SF), have emerged as promising strategies to enhance meniscal healing. This review evaluates the efficiency of these biological agents in promoting meniscal repair, with a particular focus on their potential integration into injectable hydrogel systems for targeted, minimally invasive delivery. Recent literature from 2015 to 2025 has provided growing insights into the role of biologically active agents and biomaterials in meniscal repair. Among the agents studied, PRP, MSCs, and HA have shown particular promise in modulating inflammation and supporting tissue regeneration. While biological therapies alone may not replace surgery for complex tears, they offer promising, less invasive alternatives that support tissue preservation. However, variability in study design, agent quality, and treatment protocols remains challenging. Further long-term research will be essential to confirm clinical benefits and optimize hydrogel-based delivery methods.

## 1. Introduction

**Figure 1:**
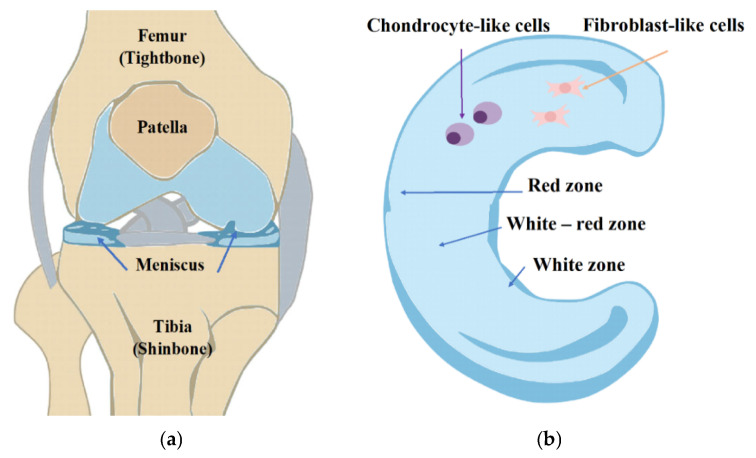
(**a**) Anatomy of the knee. (**b**) Regional variations in vascularization and cell populations of the meniscus, pointing out the vascular and avascular regions of the meniscus.

The human organism is a highly integrated and complex structure where multiple tissues function together to provide structural stability and physiological balance. Of its key functional systems, the musculoskeletal system, including bone, muscle, connective tissues like tendons, ligaments, and joints, is crucial in movement and mechanical stability (Figure 1a) [1]. Injuries to osteoarticular tissues are a multidisciplinary challenge that requires synergistic combinations of clinical experience, biological understanding, and engineering approaches to develop effective and long-lasting repair.

In recent years, injectable biological treatments have been studied as new solutions for regenerating damaged tissue, driven by advancements in tissue engineering and biomaterial science [2,3]. They are designed to repair structural damage and restore biological function by exploiting the body’s healing potential. Synovial rasping, creation of vascular access channels, platelet-rich plasma injections, and fibrin glue have been applied to promote healing and integration in challenging tissue environments like meniscus. Biological agents contribute to meniscal repair through distinct mechanisms. PRP provides a rich reservoir of growth factors stimulating angiogenesis and tissue repair [1]. MSCs exhibit multilineage differentiation and immunomodulatory properties, making them essential to tissue regeneration strategies [2]. HA is a viscoelastic component that enhances joint lubrication and cellular migration [3], while BMAC delivers a heterogeneous population of progenitor cells and growth factors directly from bone marrow [4]. Collagen provides a natural extracellular matrix scaffold supporting cell adhesion and remodeling, while exogenous GFs stimulate proliferation and differentiation directly [5]. More recently, SF has been recognized as a versatile biomaterial offering improved mechanical strength, biocompatibility, and sustained release of therapeutic agents [6]. While these agents present favorable properties, the injectable hydrogels provide an advantageous platform for delivering them due to their minimally invasive application, ability to adjust to irregular defects, and potential for controlled release and local retention. However, challenges remain, including batch-to-batch variability, rapid clearance, limited mechanical strength, temperature gelation, and difficulties achieving controlled and on-place release. These advantages and limitations highlight the potential of injectable hydrogels while underlining the need for optimized formulations to achieve strong therapeutic outcomes.

Meniscal injuries are common, often resulting from sports activities, trauma, or degenerative changes in older populations. The meniscus’s unique anatomy consists of three zones, which are differentially vascularized (Figure 1b); thus, a healing response is highly contingent upon the site zone of the tear. Tears in the outer, vascularized zone are more likely to heal, while those in the inner, avascular zone are notoriously resistant to repair [4]. The conventional treatments for meniscal injury are partial or total meniscectomy, and these can relieve symptoms but raise the risk of eventual degeneration of the joint [5,6]. Therefore, there has been a developing interest in tissue engineering techniques that attempt to preserve meniscal tissue and promote its regeneration.

**Figure 2:**
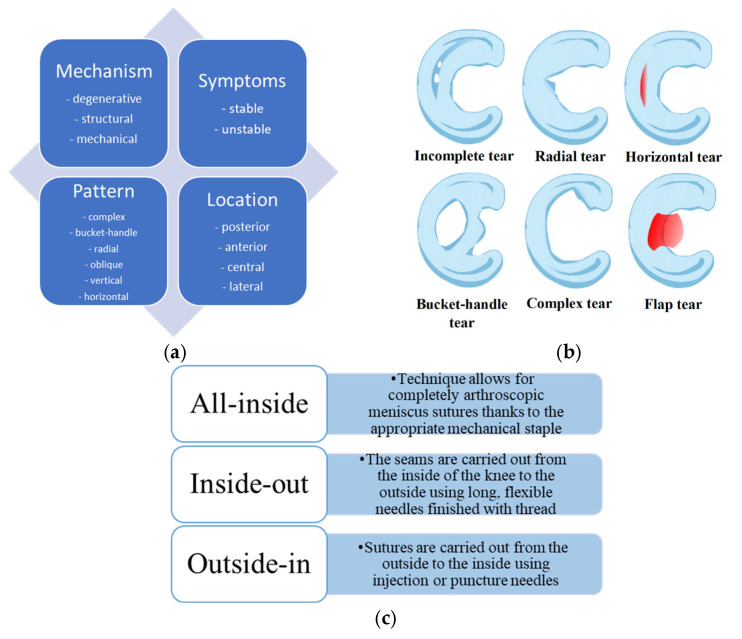
(**a**) Schematic presentation of meniscus damage classification. (**b**) Examples of the most common meniscus tears. (**c**) Basic techniques of arthroscopic operation procedures.

Meniscus damage can be classified into different categories regarding the damage mechanism, symptoms, pattern, and location (Figure 2a). Depending on the symptoms, injuries are divided into stable, where no surgical treatment is required, and unstable, where long-term damage to the meniscus may lead to injury to the articular cartilage, which may require surgical treatment. Lesions are classified according to the harm’s location and are divided into the zone supplied with blood (red zone) or not supplied with blood (white zone). The posterior horn of the medial meniscus is the most commonly damaged. Its characteristic symptoms are pain when squatting and performing rotational movements. Lastly, meniscus tears can be classified according to the pattern, including vertical longitudinal, oblique, radial, horizontal, and complex (including degenerative) tears (Figure 2b) [7]. Depending on the damage, basic methods of arthroscopic operation procedures for meniscus tears have been developed (Figure 2c). Advances in biological augmentation, particularly when paired with injectable hydrogel systems, have brought new possibilities for repairing meniscal injuries once considered irreparable [8]. These advances aim to create a biologically favorable microenvironment conducive to cell viability, growth factor retention, and tissue integration at the injury site.

Key biological agents investigated for meniscal repair include hyaluronic acid (provides lubrication and reduces inflammation), platelet-rich plasma (delivers concentrated growth factors such as PDGF and TGF-β), mesenchymal stem cells (stimulate regeneration and modulate immune response), collagen (biomimetic scaffold for extracellular matrix support), and silk fibroin (mechanically robust, biodegradable scaffold material). Unlike previous reviews that addressed biological augmentation or biomaterials separately, the novelty of this work lies in gathering information on injectable hydrogels as delivery matrices for these biological agents. This integrated perspective highlights the translational potential of combining bioactive compounds with engineered delivery systems, offering clinicians insight into minimally invasive meniscal preservation and repair strategies.

This review aims to present an up-to-date overview of the most commonly used biological augmentations for meniscus repair, emphasizing the therapeutic potential of injectable hydrogels. The following key questions are addressed to guide the discussion: 1. What are the most common biological augmentations currently used in managing meniscal injuries? 2. How do different tear types and locations affect the success of biological therapies? 3. How do injectable hydrogel-based treatments compare with conventional surgical repair methods? 4. What are the major challenges and limitations of current injectable biological therapies in clinical settings? By addressing these questions, this review seeks to contribute to the broader understanding of regenerative approaches in meniscal repair and highlight promising directions for future research, clinical application, and biomaterial innovation.

## 2. Materials and Methods

A range of studies were selected and categorized according to the biological augmentation strategy, including injectable systems such as platelet-rich plasma, mesenchymal stem cells, hyaluronic acid, and silk fibroin-based hydrogels. Particular attention was given to studies that utilized injectable hydrogels as delivery systems or carriers for these biological treatments. Each study was reviewed for clinical relevance and methodological quality, with specific interest in randomized controlled trials (RCTs) and high-quality preclinical or experimental investigations offering insight into the regenerative potential of hydrogel-based therapies for meniscal repair.

Full-length, peer-reviewed publications in English from 2015 to 2025 were considered to provide a comprehensive overview. Exclusion criteria were conference abstracts, non-English articles, and studies unrelated to meniscal tissue repair. Literature searches were conducted using databases such as ScienceDirect, PubMed, and Google Scholar, applying search terms including “osteoarthritis,” “biomaterial,” “meniscus repair,” and “biological.” Studies were selected based on relevance, originality, and alignment with the topic of injectable biological therapies. In total, 70 articles were included in this review. These studies were categorized by the type of biological augmentation used, the nature of the meniscal tear addressed, the injectable biomaterial applied, and reported clinical or experimental outcomes.

## 3. Results

The most commonly used biological augmentations for meniscal injuries include hyaluronic acid [9], platelet-rich plasma [10], mesenchymal stem cells, bone marrow aspirate concentrate [11], collagen, and silk fibroin [12]. HA is the most widely applied natural polymer, valued primarily for its lubricating and shock-absorbing properties. It has demonstrated effectiveness in treating osteoarthritis and improving the healing environment within the knee joint. PRP, containing various growth factors such as platelet-derived growth factor (PDGF), vascular endothelial growth factor (VEGF), and transforming growth factor-beta (TGF-β), has shown considerable promise in stimulating tissue repair by promoting cell proliferation and enhancing the overall healing process [13]. MSCs, often administered with chondrogenic factors [14,15], contribute significantly to tissue regeneration by stimulating chondrocyte metabolism and modulating inflammation typically observed in osteoarthritis [16]. BMAC, utilizing stem cells derived from the patient’s bone marrow, has demonstrated the capacity to generate meniscus-like tissue, supporting meniscal repair and integration with surrounding tissues [17,18,19]. Collagen, particularly type I collagen, is frequently employed as a scaffold in tissue engineering due to its structural compatibility with the meniscus extracellular matrix [20,21]. Similarly, silk fibroin is noted for its high biocompatibility and biodegradability, making it suitable for constructing scaffolds and hydrogels designed to promote tissue regeneration [22,23].

These biological agents are often used individually but can also be combined with other materials to enhance therapeutic outcomes. Several studies have demonstrated improved healing when HA, PRP, and MSCs are incorporated into scaffold systems, underscoring the potential benefits of combinatory approaches (Table 1).

Recent advances in biological augmentation for meniscus repair have focused on biological agents combined with biomaterial matrices. PRP, a rich autologous source of growth factors such as PDGF, VEGF, TGF-β, and FGF [28,29,30], has shown to enhance chondrocyte and MSC proliferation, modulate inflammation, and promote ECM deposition [31,32,33,34], with innovations such as photo-cross-linkable PRP hydrogels [31], silk fibroin–PRP scaffolds [35], and multifunctional PRP–chitosan composites [37] significantly extending its regenerative scope. HA, a key ECM glycosaminoglycan, contributes to lubrication and viscoelasticity [20], with hybrid scaffolds and hydrogels functionalized with nanoparticles, peptides, or curcumin showing enhanced biomechanical, antimicrobial, and chondrogenic effects in preclinical models [21,22,23,24]. However, clinical evidence remains mixed, as two-year follow-up studies reported no significant meniscus regeneration after HA injection alone [26]. Growth factor therapies, including TGF-β, FGF, IGFs, and BMPs, directly stimulate proliferation, ECM synthesis, and angiogenesis, with combinatorial hydrogel systems, such as CTGF–TGF-β3 staged-release constructs, achieving superior fibrocartilaginous healing compared with single-factor approaches [45,49]. MSCs and BMAC provide potent regenerative capacity, with synovial MSCs demonstrating powerful proliferative potential in animal and human studies [55,56,57,58,59,60,61,62,63,64,65]. Injectable scaffolds based on HA, PEG, or chitosan further intensify MSC chondrogenesis [22,66,67,68,69]. In contrast, BMAC-enhanced scaffolds such as decellularized meniscus ECM or silk fibroin hydrogels promote superior fibrochondrogenic differentiation, ECM deposition, and OA prevention compared with conventional matrices [67,68]. Although central to meniscus structure, Collagen suffers from weak mechanics and rapid degradation. Still, its bioactivity can be augmented by biochemical modifications, such as CS incorporation [48], collagen-binding peptides, or cross-linking strategies with riboflavin [52], emerging bioactive additives like holomycin, or suppressing inflammation while promoting collagen II synthesis [21]. Fibrin clots, finally, offer a simple, low-cost alternative to PRP, delivering structural support and platelet-derived growth factors [30], with both animal and clinical studies reporting promising outcomes, from improved meniscal regeneration in rabbit models [65] to 75% healing rates in degenerative tears.

The location and type of meniscal tears significantly influence the effectiveness of these biological treatments. Meniscal tears are classified as horizontal, vertical, radial, or complex, with healing potential varying according to tear location. Tears in the avascular inner zone of the meniscus are particularly challenging to treat due to the lack of blood supply necessary for healing. Consequently, biological therapies such as PRP and MSCs have shown limited success in this zone. In contrast, treatments applied to the vascularized outer zone are more effective, benefiting from improved blood flow that supports tissue regeneration. Research by Zhong et al. and Ozeki et al. demonstrated that MSCs combined with scaffolds or growth factors stimulate tissue repair more effectively in the outer meniscus [1,46]. Likewise, BMAC, silk fibroin, and collagen scaffolds have enhanced tear repair in more vascularized areas [56]. Despite these advances, optimizing biological therapies for avascular tears remains a significant challenge, emphasizing tear location as a critical factor in treatment success [57].

While traditional surgical approaches such as meniscectomy and meniscus repair continue to play an important role, biological therapies offer unique advantages in promoting tissue regeneration and preserving meniscal function. Surgical interventions often involve the removal or suturing of damaged meniscal tissue, which can lead to joint instability, chronic pain, and increased osteoarthritis risk over time. Conversely, biological therapies like MSCs, PRP, and BMAC aim to regenerate damaged tissue while maintaining the native meniscal structure and function. Evidence suggests that combining biological agents with scaffolds or extracellular matrices may produce synergistic effects, resulting in enhanced tissue regeneration and superior structural integrity compared to meniscectomy alone. For example, Zhong et al. reported that BMAC combined with decellularized meniscus scaffolds achieved superior healing compared to conventional surgical repair [40,46]. Additionally, HA and PRP reduce inflammation and improve joint lubrication, supporting recovery and long-term joint function [58,59]. Despite promising early clinical outcomes, larger-scale, long-term comparative studies are required to fully evaluate biological therapies’ safety, efficacy, and cost-effectiveness relative to traditional surgical methods.

While each biologically active agent offers distinct regenerative benefits, their integration into injectable hydrogel matrices represents the most promising and clinically adaptable approach for meniscal repair. Combinatory strategies, such as MSCs in PRP-enriched hydrogels or HA-functionalized scaffolds, can synergistically enhance biological activity, mechanical support, and clinical feasibility. Key parameters include degradation rate, which must balance resorption with structural support to ensure durability in load-bearing regions and injectability and gelation kinetics, allowing minimally invasive delivery and in situ stabilization. However, biological augmentation holds promise for wider clinical use, but several obstacles remain. One major challenge is the variation in protocols for application, such as PRP preparation, HA formulations, and MSC dosages, which influence therapy outcomes. For example, Olesen et al. showed that leukocyte-rich PRP may yield different results than leukocyte-poor PRP, where using specific centrifugation protocols or filtration systems to selectively retain or remove leukocytes, resulting in leukocyte-rich PRP (LR-PRP) or leukocyte-poor PRP (LP-PRP), which differ in their inflammatory and regenerative properties [37]. PRP is most commonly prepared by single- or double-spin centrifugation of autologous blood, achieving platelet concentrations three to five times above complete blood. Before clinical use, it can be activated with calcium chloride or thrombin, which induces platelet degranulation and enhances growth factor release. Another limitation is the difficulty of treating meniscus tears in the avascular zone, where biological treatments struggle due to a lack of blood supply. While MSCs and PRP offer some benefits, their effectiveness in the inner meniscus zone remains unclear [60,61]. Interestingly, the amount of leukocytes present in PRP is essential, as preparations of PRP low in leukocytes promote better tissue integration. On the other hand, therapies based on MSCs generally produce stronger regenerative responses, particularly in vascularized meniscal tears and when combined with scaffold materials. HA-based hydrogels mainly offer temporary symptom relief rather than actual structural healing. So, it becomes clear that the type of meniscal tear, blood supply, and even the patient’s age must be considered when deciding on treatment. The optimal delivery method for biological therapies, whether injectable formulations, scaffolds, or hydrogels, is still under investigation, with multiple studies evaluating the best combinations for healing efficacy. The high cost of biological treatments, including scaffolds and biomaterials, limits patient access and availability. However, MSC treatments and scaffold-related hydrogels are less cost-effective than traditional meniscectomies. Their practical value likely hinges on whether they delay osteoarthritis progression or reduce the need for joint replacements over time. Therefore, injectable hydrogels are promising because they enable minimally invasive delivery, can release bioactive molecules locally, and may improve tissue integration. However, problems like insufficient mechanical strength, slow or inconsistent gelation, and high production costs still stand in the way of broad clinical use. Table 2 presents an overview of the current biological additives and hydrogel-based methods used for meniscal repair, categorized by their sources, delivery modes, benefits, drawbacks, and observed outcomes.

## 4. Discussion

This review highlights the various biological augmentations used in treating osteoarticular injuries, focusing on the controversy surrounding different types of meniscus injuries and their treatment. The type of meniscus injury significantly impacts the treatment approach, particularly the use of biologicals and biomaterials.

Biological augmentations have been used to repair meniscus lesions, such as hyaluronic acid, for actual lubrication and protection of the joints. Hyaluronic acid (HA) is widely used for joint lubrication and protection. Research suggests that enhancing HA’s properties, such as creating injectable cartilage-coating composites incorporating decellularized extracellular matrices (dECMs), can provide prolonged joint protection and superior lubrication [43,44]. Moreover, HA-based hydrogels delivering bioactive molecules have demonstrated anti-inflammatory effects and improved chondrocyte nourishment, leading to better meniscal repair outcomes [24]. For example, HA hydrogels loaded with selenium nanoparticles have significantly improved cartilage homeostasis and symptom relief in osteoarthritis models [25]. Hybrid constructs combining HA with polycaprolactone and dECMs have also demonstrated promise in repairing partial meniscal injuries [26].

PRP is yet another promising type of biological augmentation. Results have shown that PRP can induce chondrogenesis, inhibit inflammation, and promote the migration and proliferation of chondrocytes and mesenchymal stem cells, thus contributing to cartilage regeneration [28,29,30]. PRP combined with silk fibroin bio-ink prepared an SF-PRP scaffold, improving biological properties and biocompatibility for cartilage repair [53,54,62]. However, a few studies have shown that repeated injections of PRP fail to produce consistent results regarding macroscopic and histological changes observed for cartilage repair [34,35,63].

Growth factors, such as platelet-derived growth factors, have also been used in treating meniscus injuries. PDGF-coated decellularized meniscus scaffolds have been shown to promote meniscus cell migration and improve defect healing [37]. These growth factors are believed to counteract the effects of inflammatory cytokines such as IL-1, which can hinder meniscal repair by MMPs that degrade collagen [36]. Meniscus injury type significantly impacts the choice and success of biological treatments. For example, tears that involve both the meniscal body and peripheral areas or those with poor vascularization may require biological augmentations that facilitate cellular migration and tissue regeneration. Studies indicate that growth factors like PDGF or combining MSCs with decellularized matrices can improve healing in these more complex tears. Furthermore, mesenchymal stem cells have been combined with scaffold materials such as decellularized skin matrix scaffolds (DSM) to slow osteoarthritis progression by supporting cartilage matrix production. A first-in-human study demonstrated the positive effect of synovial MSC transplantation on meniscus complex degenerative tear healing, showing improved clinical symptoms and MRI findings after two years [40,41]. In contrast, simple meniscal tears may respond more favorably to treatments such as HA injections or PRP, which provide relief and promote tissue repair without needing cell-based therapies. Nevertheless, the variability in response to biological treatments underscores the need for personalized approaches, particularly in the presence of different tear patterns and stages of degeneration.

These therapies offer patients treatment with less invasive procedures than classical surgical meniscectomy or meniscal repair procedures involving suturing or stapling. Although meniscectomy has given short-term relief, subsequent long-term degeneration and knee OA are seen. However, biological treatments regenerate meniscal tissue to maintain function while preventing OA development. Many studies comparing the types of treatments, such as PRP, HA, and growth factor therapies, with surgical interventions suggest that all the biological treatments enhance tissue repair and reduce inflammation. For example, PRP and HA significantly enhance cartilage repair and reduce inflammation in OA models, while other growth factors, such as PDGF, contribute to meniscal tissue regeneration [37,38].

Furthermore, MSC-based therapies have been in the limelight for their potential to regenerate meniscus tissue and facilitate healing. Clinical studies showed that MSCs with scaffolds such as decellularized meniscus matrices or synthetic hydrogels restore meniscal structure and function more effectively than traditional surgery alone [38,39]. Such treatments have, in some cases, been found to be higher in healing and conservation of cartilage in the long term; however, the other side of the coin is that the efficacy of such biological therapies is conditioned in most cases on treatment protocols, type of injury, and patient factors. Though alternative biological therapy shows promise, a broader validation through randomized controlled trials still needs to be performed to define its practice in the clinical scenario [39].

Biological augmentations manifest several difficulties in their application to meniscus repair, one such being that prosthetic agents vary in efficacy among patients, as they might prove age, activity level, and type of tear dependent. More specifically, HA and PRP may improve tissue repair, but they have diminished effectiveness when used for severe or chronic degeneration [25,27,35]. The lack of standards for the preparation and application of PRP thus leaves room for significant variation in clinical outcomes and complicates the interpretation of study results in the area [64]. One of the other limitations is the definite lack of research on the long-term results of these biological treatments. While promising results have been noted from preclinical and small-scale trials, the long-term viability of biological therapies for the repair of the meniscus remains in question. Additional research is still needed to determine the regenerative potential of these therapies, such as PRP, HA, and MSCs, particularly concerning their long-term protection from developing OA [65,66].

Several ongoing clinical efforts are attempting to harmonize protocols, which may help achieve reproducible results across centers. Beyond biological effectiveness, economic feasibility remains a barrier to widespread clinical adoption. Injectable hydrogels allow minimally invasive administration, localized release of bioactive factors, and improved tissue integration while preserving surrounding structures. However, disadvantages include insufficient load-bearing strength under repetitive mechanical stress, limited efficacy in avascular zones, variability in gelation kinetics, and high production costs. These trade-offs must be addressed through material optimization and standardized protocols. Scaffolds, therefore, create additional challenges for meniscus injury repairs since an ideal scaffold material should exhibit biocompatibility, mechanical support, and efficient tissue regeneration. Researchers are improving scaffold structures such as silk fibroin and collagen scaffolds. However, all should still pass the tests for clinical use, such as regarding degradation rates and integrative ability with the native meniscus tissue [67].

## 5. Conclusions and Future Directions

This review emphasizes the translational potential of combining biological augmentation strategies with injectable hydrogel delivery systems. Hydrogels represent enabling platforms that connect regenerative therapies with minimally invasive surgical techniques. Future research should prioritize biomaterial optimization, cost-effectiveness analyses, protocol standardization, and long-term clinical trials to confirm durability and clinical utility in meniscal repair.

Substantial variability in the literature for each biological augmentation method makes comparisons difficult. Nevertheless, recent clinical trials have shown beneficial results. Biological additives such as silk fibroin, hyaluronic acid, platelet-rich plasma, and bone marrow aspirate have demonstrated effectiveness or advantages compared to conventional meniscus repair. These clinical trials have also confirmed the safety of biological treatments. However, the lack of clarity regarding efficiency may result in patients and healthcare systems undergoing unnecessary surgeries and incurring high costs. Despite this, biological enhancements have shown promise by promoting the ability of damaged tissue to repair and regenerate itself. Repairing meniscus tears and preserving meniscal function to prevent cartilage degeneration post-surgery remain essential goals.

More research is required to gather evidence on how meniscal repair may prevent knee OA in the long run. Recently, there have been promising results in using natural polymer-based hydrogels for 3D-bioprinting to replace damaged meniscus in the future. New bio-inks can be investigated to improve cartilage and bone regeneration in laboratories and live experiments. [66]. The research primarily focuses on perfecting the bio-ink for the 3D-printed scaffolds to replace damaged meniscus. However, exciting research is ongoing on thermosensitive hydrogels called Pluronic. They are promising synthetic polymers whose sol-to-gel state is visible at higher temperatures. Several recent studies have been conducted using Pluronic F127 (poloxamer 407). Zang et al. have been working on gels based on Pluronic F127, a glycosaminoglycan-based injectable hydrogel with hyaluronic acid and chondroitin sulfate, which provides a new strategy for OA relief, hence a promising drug delivery system [68,69,70]. In another laboratory group, Meng et al. have been working on poloxamer 407 and hyaluronic acid (P-HA) injectable and thermo-responsive drug delivery systems to durably retain rapamycin and dramatically magnify their therapeutic effects in OA management. [65]. Therefore, this strategy significantly decreased the pro-inflammatory cytokines (e.g., TNF-α, IL-1β, and IL-6) and increased the anti-inflammatory cytokines (e.g., IL-10) expression in the synovial tissues and synovial fluid, inducing cartilage matrix formation, which suggests excellent potential for the management of OA.

## Figures and Tables

**Table 1:** Summary of Biological Additives for Meniscus Repair.

Biological Additive	Material	Cells	Animal Model	Effects/Outcomes	Ref.
*Hyaluronic acid*	Decellularized cartilage matrix (dECMs) crosslinked by hyaluronan composite	Chondrocytes	Male SD rats	Increased cell viability and ECM deposition, improved joint lubrication	[24]
	Aldehyde-modified HA (HAA) gel crosslinked by carbamate-modified polyvinyl alcohol (PVAC)	-	C57BL/6 mice	Rapid in situ gelation, reduced inflammation	[25]
	Hydroxypropyl chitin (HPCH)/hyaluronic acid hydrogel	Primary chondrocytes, BMSCs	C57BL/6J mice	Sustained release of HA, increased proteoglycan synthesis	[26]
	Oxidized hyaluronic acid (OHA) with adipic dihydrazide-grafted HA (HA-ADH) solution and selenium nanoparticles (SeNPs) hydrogel	SW1353 cells	Male SD rats	Antioxidant effect, decrease MMP expression	[27]
	Polycaprolactone (PCL) and decellularized meniscus extracellular matrix (DMECM) surface modified by gelatin (G), hyaluronic acid (HU), and selenium (Se) nanoparticles (PCL/DMECM/G/HU/Se)	Chondrocytes and adipose-extracted mesenchymal stem cells (ASCs) obtained from Hoffa’s fat pad	The medial meniscus of the right knee in the rabbit model	Increase mechanical stiffness, enhanced fibrocartilage formation	[28]
	Fibrin-hyaluronic acid hydrogel	Human articular chondrocytes	-	Injectable, promotes cell migration	[29]
	Hyaluronic acid and basic fibroblast growth factors (bFGF)	Chondrocytes and synovial cells	Japanese white rabbits	Increased cell proliferation, faster defect closure	[30]
*Platelet-rich plasma*	An in situ photocrosslinkable PRP hydrogel glue (HNPRP)	Human chondrocyte and L929 mouse fibroblast cells	New Zealand rabbits	Rapid gelation, increased matrix production	[31]
	10% Calcium chloride-activated PRP	Bone marrow-derived mesenchymal stem cells (BMSCs)	Mature male rabbits	Enhanced osteogenic differentiation	[32,33]
	Silk-fibroin with PRP scaffolds (SF-PRP) (50% PRP, *v*/*v*)	The rabbit chondrocytes	New Zealand white rabbits	Increased tensile strength, improved healing	[34,35]
	Black phosphorus nanosheets (BPNs)- platelet-rich plasma (PRP)-chitosan (BPNs/Chitosan/PRP)	RAW264.7 cells, L929 cells, and MSC cells	DBA1/J mice	Antibacterial and pro-regenerative properties	[36]
	Bone marrow stimulation (BMS) with activated PRP	Fibrocartilage cells	Sheep and minipig model	Increased fibrocartilage formation	[37]
*Growth factors*	Cytokine interleukin-1 (IL-1) in matrix metalloproteinase (MMP)	Meniscal cells	Murine model	Increased cell migration, ECM synthesis	[38]
	Platelet-derived growth factor (PDGF)-coated decellularized meniscus scaffold (DCM)	Human avascular meniscus cells	-	Increased cell migration, ECM synthesis	[39]
*Mesenchymal stem cells*	MSCs cultured in PRP	Articular chondrocytes	Rat model	Increased chondrogenic markers	[15,40]
	Decellularized skin matrix with PFSSTKT (PFS) peptide and mesenchymal stem cells	Murine fibroblasts (L929), rat adipose-derived mesenchymal stem cells (ADSCs)	New Zealand White rabbit	Increased cell infiltration, ECM deposition	[41]
	Transplanted autologous synovial MSCs	Synovial MSCs	Human	Clinical improvement, MRI-confirmed repair	[42]
	Synovial mesenchymal stem cells	Synovial MSCs	Minipig models	Robust tissue integration	[43,44]
*Bone Marrow Aspirate*	Bone Marrow Aspirate Concentrate	-	Human	Generation of meniscus-like tissue, integration	[19]
	Decellularized meniscus extracellular matrix (mECM) hydrogel	Adult mesenchymal stem cells	An SD rat model	Increased fibrocartilage formation	[45]
	Silk fibroin-MSCs porous scaffolds	Bone marrow mesenchymal stem cells	New Zealand white rabbits	Mechanical reinforcement and regeneration	[46]
	GC/4- arm PEG-CHO hydrogel	Bone mesenchymal stromal cells (BMSCs)	New Zealand White rabbits	Controlled release, improved healing	[47]
	Chondroitin sulfate succinimide succinate (CS-NHS) and bone marrow aspirate hydrogels (CS-BM)	Meniscus fibrochondrocytes	The athymic rat model and the rabbit femoral defect model	Increased matrix deposition, reduced OA	[48,49]
*Collagen*	Human type I collagen combined with autologous platelet-rich plasma (STR/PRP)	Fibroblast	Human	Increased proliferation and matrix production, enhanced healing response	[20]
	Collagen type I (Col I) and activated chondroitin sulfate hydrogels	Chondrocytes	SD rats	Promoted chondrogenesis, increased ECM synthesis and cartilage-like tissue repair	[50]
	Collagen with poly (lactic-co-glycolic acid) (PLGA) microparticles hydrogel	Human umbilical vein endothelial cells (GFP-HUVECs)	-	Supported angiogenesis, increased cell adhesion and viability	[51]
	Riboflavin-induced photo-crosslinking of collagen hydrogel	Fibrochondrocyte cells	New Zealand white rabbit	Increased biomechanical strength, improved tissue integration	[52]
	Holomycin (HL)	Murine primary chondrocytes	OA mouse model	Anti-inflammatory effects, protected cartilage matrix	[21]
*Silk fibroin*	Silk fibroin and gelatin methacrylate with encapsulated platelet-rich plasma (PRP)	BMSCs, chondrocytes,	SD rats	Increased chondrogenic differentiation, promoted cartilage repair	[53]
	Glycidyl methacrylate (GMA)-modified silk fibroin hydrogel	BMSCs	New Zealand rabbits	Enhanced scaffold elasticity, supported cartilage regeneration	[54]
	Silk fibroin hydrogel crosslinked by diglycidyl ether (BDDE)	Rat bone mesenchymal stem cells	Male SD rats	Improved mechanical stability, increased cartilage-like ECM deposition	[55]
	Silk fibroin/gelatin methacrylate hydrogel	BMSCs	Male SD rats	Supported cell proliferation and chondrogenesis	[53]

**Table 2:** Summary of composition, delivery, and clinical outcomes of biologic and hydrogel-based meniscal therapies.

Biological Additive/Hydrogel	Source/Composition	Delivery Method	Advantages	Limitations	Key Results/Outcomes
*PRP*	Autologous platelet concentrate	Direct injection/combined with scaffold	Rich in growth factors, easy preparation	High variability in preparation, short-term activity	Enhanced chondrogenesis, improved short-term healing, and inconsistent long-term results
*MSCs*	Bone marrow/adipose/synovial tissue	Injection/hydrogel encapsulation	Differentiation potential, immunomodulation	Regulatory and safety concerns	Improved healing rates in animal studies, increased matrix deposition
*Hyaluronic Acid (HA) Hydrogels*	Natural polysaccharide	Injectable hydrogel	Biocompatible, lubrication, chondroprotective	Weak mechanical properties	Improved lubrication, reduced inflammation, and early clinical benefits
*Collagen Hydrogels*	Type I/II collagen	Injectable hydrogel/scaffold	Good biocompatibility, a scaffold for cells	Potential immunogenicity	Promoted cell adhesion, enhanced tissue integration
*Silk Fibroin*	Natural protein (silkworms)	Hydrogel/scaffold	Strong mechanical properties, tunable biodegradability	Processing complexity	Provided mechanical reinforcement, supported meniscal regeneration

## Data Availability

The data presented in this study are available on request from the corresponding author.
